# High-Fidelity Synthesis of Temporomandibular Joint Cone-Beam Computed Tomography Images via Latent Diffusion Models

**DOI:** 10.3390/jcm15093344

**Published:** 2026-04-28

**Authors:** Qinlanhui Zhang, Yunhao Zheng, Jun Wang

**Affiliations:** State Key Laboratory of Oral Diseases, National Centre for Stomatology and National Clinical Research Centre for Oral Diseases, West China Hospital of Stomatology, Sichuan University, Chengdu 600041, China

**Keywords:** temporomandibular joint, cone-beam computed tomography, latent diffusion models, generative artificial intelligence, confidentiality

## Abstract

**Background**: The development of robust artificial intelligence (AI) models for diagnosing Temporomandibular Disorders (TMDs) is severely constrained by data scarcity and patient privacy regulations. Cone-beam computed tomography (CBCT), the gold standard for assessing osseous changes in the temporomandibular joint (TMJ), inherently contains sensitive biometric facial features, making de-identification difficult without losing critical anatomical information. This study aims to develop and evaluate TMJCTGenerator, a specialized latent diffusion model (LDM) framework designed to synthesize high-fidelity, diverse, and anonymous TMJ CBCT images. We hypothesize that this LDM approach can achieve superior anatomical fidelity and diversity compared to traditional generative adversarial network (GAN)- and variational autoencoder (VAE)-based methods, specifically in capturing fine osseous details within sagittal and coronal views of the mandibular condyle. **Methods**: A training dataset comprising 348 anonymized CBCT volumes was obtained in this retrospective comparative study to extract high-resolution sagittal and coronal regions of interest of the mandibular condyle. An independent test set of 39 anonymized CBCT volumes was further included. We developed a class-conditional LDM that integrates a pre-trained VAE for perceptual compression with a conditional U-Net for iterative denoising in the latent space. Performance was evaluated via qualitative anatomical fidelity assessment, Fréchet Inception Distance (FID), and a blinded Visual Turing test conducted by experienced clinicians to determine the distinguishability of synthetic images from real data. **Results:** Qualitative analysis revealed that TMJCTGenerator produced images with superior sharpness and anatomical consistency compared to baseline models, successfully reconstructing fine bone structures essential for diagnosing degenerative joint disease. TMJCTGenerator achieved lower FID scores than both VAE and GAN baselines. In the visual Turing test, clinicians were unable to reliably distinguish the generated images from real scans, and non-inferiority analysis confirmed that the synthetic data were statistically non-inferior to real data. Furthermore, TMJCTGenerator demonstrated the capability to generate diverse pathological conditions, ranging from normal anatomy to severe osteoarthritic changes. **Conclusions**: The proposed LDM framework effectively addresses the data scarcity and privacy bottlenecks in TMJ AI research by generating realistic, fully anonymous medical imaging data. TMJCTGenerator outperforms traditional generative methods in both visual fidelity and diversity, offering a viable solution for training downstream diagnostic algorithms. The source code and pre-trained models of TMJCTGenerator have been made open-source.

## 1. Introduction

Temporomandibular disorders (TMDs) encompass a group of musculoskeletal and neuromuscular conditions involving the temporomandibular joint (TMJ), the masticatory muscles, and all associated structures [1]. These disorders affect approximately 34% of the global population and significantly impact patients’ quality of life [2,3,4]. Accurate diagnosis and treatment planning for TMDs rely heavily on medical imaging [5]. Cone-beam computed tomography (CBCT) has established itself as the diagnostic gold standard for assessing osseous changes associated with degenerative joint disease (DJD) and osteoarthritis (OA) [5]. CBCT offers high-resolution, three-dimensional visualization of the mandibular condyle and glenoid fossa, enabling clinicians to detect subtle bony erosions, osteophytes, and flattening that are critical for early diagnosis [6,7,8]. Furthermore, CBCT provides indirect indicators of disc displacement, such as widened anterior joint space [6,9].

Despite the clinical importance of CBCT, the development of robust, data-driven artificial intelligence (AI) models for automatic TMD diagnosis is severely hindered by data scarcity and privacy concerns, compounded by the lack of public TMJ image datasets [10]. Deep learning algorithms require massive, diverse datasets to generalize effectively [11]. However, constructing large-scale, public CBCT datasets is challenging due to strict patient privacy regulations. Unlike other medical modalities, head and neck CBCT scans inherently contain sensitive biometric facial features, making patient re-identification a trivial task. Traditional de-identification methods, such as skull stripping or facial defacing, often involve cropping the volume below the nasal floor to isolate dental anatomy [12,13]. Unfortunately, this approach inevitably results in the exclusion of the TMJ structures [12,13]. Consequently, the scarcity of high-quality, privacy-preserved public datasets remains a primary bottleneck in TMJ AI research.

Generative AI offers a promising solution to this challenge by synthesizing realistic, anonymous medical images that preserve the statistical properties of the original population without exposing individual patient data [14,15]. In recent years, generative adversarial networks (GANs) and variational autoencoders (VAEs) have been the dominant approaches for medical image synthesis [16,17,18]. However, both architectures face inherent limitations. GANs, despite their ability to generate sharp images, are notoriously difficult to train and often suffer from “mode collapse,” where the model generates a limited variety of samples, failing to capture the full spectrum of anatomical variability [19]. Conversely, VAEs offer stable training and good diversity but typically produce blurred images with a loss of high-frequency details, such as the fine trabecular bone structure essential for DJD diagnosis [20].

To overcome these limitations, denoising diffusion probabilistic models, and specifically latent diffusion models (LDMs), have emerged as the new state of the art in generative modeling [21]. LDMs combine the perceptual power of VAEs with the iterative refinement of diffusion processes, allowing for the synthesis of high-fidelity images with computationally efficient training in a compressed latent space [21].

Generative models, particularly LDMs, have been extensively applied to general medical imaging modalities such as X-ray, CT, and MRI [22], demonstrating strong performance in specialized tasks like cephalometric synthesis [23]. Conversely, the TMJ field currently suffers from a dearth of both generative models and public datasets. This scarcity creates a significant research bottleneck, as the lack of standardized benchmarks precludes the objective comparison of AI algorithms. Consequently, the advancement of robust diagnostic tools for TMD remains severely constrained.

To address the challenges of data scarcity and privacy in TMD diagnostics, this study proposes and evaluates TMJCTGenerator, a specialized LDM framework. We hypothesize that the proposed LDM can generate TMJ CBCT images that are superior in both anatomical fidelity and diversity compared to traditional GAN- and VAE-based approaches, particularly for high-resolution sagittal views of the mandibular condyle.

Our contributions are as follows:

1. We develop TMJCTGenerator, an LDM specifically tailored for TMJ CBCT synthesis, capable of reproducing fine osseous details indicative of osteoarthritic changes.
2. We provide a comparative analysis of other methods and conduct a blinded visual Turing test with dental experts to evaluate the clinical realism of the generated images against real scans.
3. To promote reproducibility and accelerate research in the TMJ AI community, we open-source our complete code implementation and pre-trained model weights at https://github.com/Zheng-Yunhao/TMJCTGenerator (accessed on 24 February 2026).

## 2. Materials and Methods

### 2.1. Dataset Acquisition and Preprocessing

This retrospective observative study was conducted in accordance with the Declaration of Helsinki and approved by the Institutional Review Board of West China Hospital of Stomatology, Sichuan University (approval number: WCHSIRB-D-2025-106, approval date: 20 February 2025).

Raw TMJ CBCT data were obtained from West China Hospital of Stomatology, Sichuan University. The training dataset comprised 348 anonymized CBCT volumes from patients who visited the Department of TMJ from March 2025 to October 2025. To establish an independent test set, data from 39 patients who visited the clinic in November 2025 were further included.

The inclusion criteria were as follows: (1) patients presenting to the Department of TMJ at West China Hospital of Stomatology; (2) completion of a CBCT examination; and (3) provision of informed consent. The exclusion criteria included: (1) CBCT scans lacking complete coverage of TMJ anatomical structures; and (2) images characterized by blurring in the TMJ region, as determined by the consensus of two dentists. The demographic distribution of age and gender for all patients is summarized in Table 1.

All CBCT data were anonymized by removing personal identifiers from the DICOM metadata and replacing them with unique study IDs. To generate high-fidelity 2D images, slices were extracted from the 3D volumes, specifically focusing on the condylar region. The sagittal and coronal slice ranges containing TMJ structures were identified by an experienced dentist. We then extracted the central sagittal and coronal slices of the TMJ to capture the maximum cross-sectional area of the joint anatomy. Finally, the extracted regions of interest (ROIs) were cropped and resized to a resolution of 256 × 256 pixels. The final training set consists of 17,423 coronal slices and 38,092 sagittal slices, while the test set comprises 1988 coronal and 4117 sagittal slices.

### 2.2. Model Architecture

We propose a class-conditional LDM framework to synthesize TMJ CBCT images, conditioned on coronal and sagittal orientations. The framework comprises three core components:

1. A pre-trained VAE that compresses high-resolution images into a low-dimensional latent space.
2. A conditional U-Net denoising network that learns to reverse the diffusion process in the latent space.
3. A class embedding module that modulates the generation process via cross-attention mechanisms based on the input condition.

#### 2.2.1. Clinical Symptom

We utilized a pre-trained VAE with Kullback–Leibler-regularization as the perceptual compression stage. This model consists of an encoder E and a decoder D.

- Encoder: Maps the input image $x\in R^{H\times W\times 3}$ into a latent representation $z=E\left(x\right)$, where $z\in R^{h\times w\times c}$. The downsampling factor was set to f=H/h=8.
- Decoder: Reconstructs the image from the latent vector, $\hat{x}=D\left(z\right)$.

In this study, to leverage the robust feature representation learned from large-scale datasets, we froze the weights of the pre-trained VAE. This ensures that the mapping between pixel space and latent space remains stable and computationally efficient during the training of the diffusion component.

#### 2.2.2. Latent Diffusion

The core generation process occurs in the latent space. We employed a time-conditional U-Net with cross-attention mechanisms. The diffusion process is modeled as a Markov chain that gradually adds Gaussian noise to the latent vector $z_{0}$ over T timesteps, producing a sequence of noisy latents $z_{1},\ldots ,z_{T}$.

The objective of the U-Net is to reverse this process by predicting the added noise ϵ from a noisy latent $z_{t}$ at timestep t. The loss function is defined as the simplified mean squared error (MSE):

$$L_{\mathrm{LDM}}=E_{z_{0},t,\epsilon ∼N\left(0,1\right)}\left[||\epsilon -\epsilon_{\theta }\left(z_{t},t\right)|{|}_{2}^{2}\right]$$

where t is uniformly sampled from {1, …, T}. We trained separate U-Net models for sagittal and coronal views to capture the distinct anatomical features of each projection plane.

### 2.3. Training Strategy and Implementation Details

The TMJCTGenerator model was implemented using the PyTorch (version 2.8.0) framework and the Diffusers library. For comparative analysis, we utilized a conditional VAE and a conditional DCGAN as baseline models [17,24]. All experiments were conducted on a workstation equipped with an NVIDIA RTX 5090 GPU (NVIDIA corporation, Santa Clara, CA, USA). The specific hyperparameters and configuration details are summarized in Table 2. The cVAE and cDCGAN models were trained using identical learning rates, batch sizes, and total epochs as those employed for the TMJCTGenerator.

### 2.4. Model Evaluation

Two dentists with seven and three years of clinical experience, respectively, participated in a blinded visual Turing test. They evaluated a randomized mix of 50 real sagittal and coronal CBCT images from the test set and 50 generated sagittal and coronal CBCT images separately. The observers were asked to rate each image on a scale from 0 (definitely AI-generated) to 10 (definitely real) for the condyle and whole TMJ respectively.

The Fréchet Inception Distance (FID) was employed to quantitatively assess the quality and diversity of the generated images. For this evaluation, 10,000 synthetic images were sampled from the generative models and compared against the real images in the test set. To ensure a domain-specific and robust feature representation for dental imaging, we utilized an InceptionV3 architecture pre-trained on RadImageNet as the feature extractor [25].

### 2.5. Statistical Analysis

Statistical analyses were performed using R (version 4.5.2; R Foundation for Statistical Computing, Vienna, Austria). For the visual Turing test, the observer scores (0–10 grade scale) were treated as continuous variables and the Mann–Whitney U test was employed to compare the scores assigned to real versus generated images. To estimate the confidence intervals (CIs), the bootstrap method with 5000 iterations was applied. A difference of one grade was established as the threshold for the non-inferiority test.

## 3. Results

### 3.1. Qualitative Assessment

**Figure 1:**
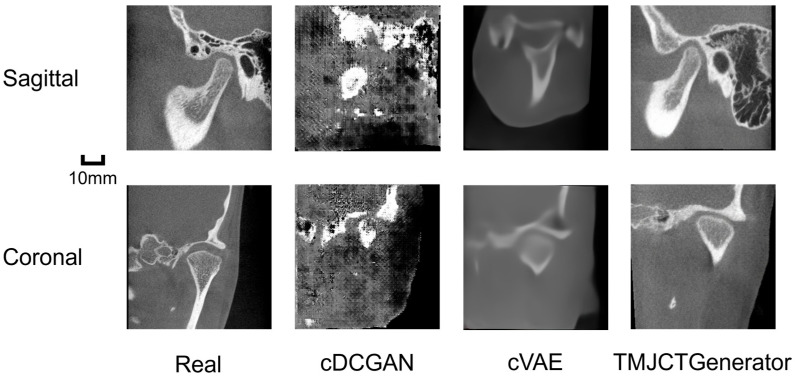
Visual comparison of real and synthesized TMJ CBCT images. The rows display representative samples in sagittal and coronal views. The columns compare real patient scans with images generated by the baseline conditional VAE (cVAE), conditional DCGAN (cDCGAN) and the proposed TMJCTGenerator. All generated samples shown were randomly drawn without quality-based selection.

Visual inspection of the synthesized TMJ CBCT images reveals distinct performance differences among the evaluated models. As shown in Figure 1, the proposed TMJCTGenerator produced high-fidelity images with sharp anatomical boundaries and realistic osseous textures in both sagittal and coronal views compared to VAE. Specifically, the LDM successfully reconstructed the delicate bone structure within the condylar head, which is critical for diagnosing early-stage degenerative changes. In contrast, the GAN model exhibited fragmented and sharp yet distorted anatomical structures under the same number of training epochs. This underscores the inherent instability and difficulty associated with training GANs for this specific task.

### 3.2. Quantitative Assessment

Table 3 presents the quantitative evaluation of the generative models using the FID. The TMJCTGenerator consistently achieved the lowest FID scores in both the coronal and sagittal views, indicating a superior ability to capture the underlying distribution of real TMJ CBCT images compared to the baseline models. While the cVAE demonstrated relatively stable performance with low FID values, it remained slightly inferior to the TMJCTGenerator. In contrast, the cDCGAN exhibited exceptionally high FID scores, reflecting its failure to generate anatomically coherent structures and suggesting significant instability during the training process for this specific task.

### 3.3. Visual Turing Test

**Figure 2:**
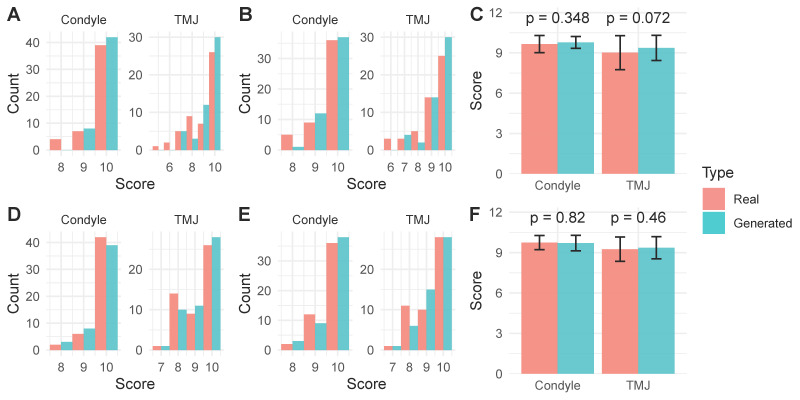
Visual Turing test for TMJCTGenerator. Realism scores were assigned on a scale of 0 to 10, where 0 represents “definitely AI-generated” and 10 represents “definitely real.” (**A**,**B**) Histogram of realism scores assigned by dentist A and B, respectively, for real versus generated sagittal CBCT images. (**C**) Comparison of mean realism scores between real and generated sagittal images for both evaluators. (**D**,**E**) Histogram of realism scores assigned by Dentist A and B, respectively, for real versus generated coronal CBCT images. (**F**) Comparison of mean realism scores between real and generated coronal images for both evaluators.

To assess the clinical realism of the generated images, a visual Turing test was conducted with two dentists. The results, visualized in the histograms in Figure 2A,B,D,E, show that the experts were unable to reliably distinguish the images generated by TMJCTGenerator from real CBCT scans. Quantitative analysis confirmed that there were no statistically significant differences in the mean realism scores assigned to real versus generated images for either the sagittal or coronal views (Figure 2C,F; *p* > 0.05). The non-inferiority analysis demonstrated that the lower bounds of the 95% CIs for the mean difference in visual Turing scores between the TMJCTGenerator and real images were all greater than the predefined margin of −1 (Table 4).

### 3.4. Diversity of Generated TMJ CBCT

**Figure 3:**
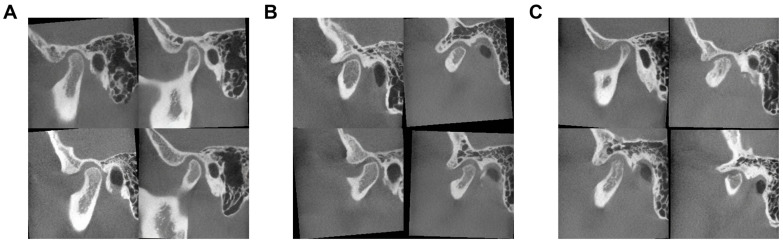
Spectrum of pathological diversity in synthesized TMJ CBCT images. (**A**) Normal TMJ showing intact cortical outlines. (**B**) Indeterminate DJD presenting with early-stage flattening. (**C**) Definite DJD characterized by severe osseous deformation.

The proposed TMJCTGenerator demonstrates an exceptional capability to capture the full spectrum of morphological variability inherent in the training population (Figure 3). The model successfully synthesizes high-fidelity images across distinct diagnostic categories, ranging from normal TMJ, characterized by smooth condylar heads and intact cortical bone, to indeterminate DJD, which displays subtle morphological deviations such as initial flattening. Furthermore, the model accurately reproduces definite DJD, exhibiting severe osteoarthritic features including distinct osteophyte formation, extensive bone erosion, subchondral cysts, and sclerosis.

## 4. Discussion

In this study, we addressed the critical challenge of data scarcity and privacy constraints in TMJ imaging by developing and validating a specialized generative framework, *TMJCTGenerator*. We hypothesized that LDMs would offer a superior alternative to traditional generative methods for synthesizing realistic medical imagery. Our findings confirm this hypothesis, demonstrating that the proposed LDM framework successfully synthesizes high-fidelity sagittal and coronal CBCT images of the mandibular condyle.

The generated images exhibited anatomical detail and textural quality significantly superior to those produced by VAEs, while avoiding the training instability observed with GANs. Furthermore, the clinical utility of the synthesized data was validated through a blinded visual Turing test, where experienced dentists were unable to reliably distinguish between real and AI-generated scans. Collectively, these results establish *TMJCTGenerator* as a robust tool for augmenting TMJ datasets, capable of capturing the full spectrum of pathological variability from normal anatomy to severe degenerative joint disease.

The superior performance of TMJCTGenerator aligns with the theoretical distinctions between generative architectures. While GANs have been explored for medical image synthesis, their application to complex osseous structures is often hampered by training instability and mode collapse, as evidenced by the failure of our GAN baseline to converge [19,26]. Conversely, VAEs ensure stability but inherently blur high-frequency details—such as the fine bone patterns critical for OA diagnosis—due to their element-wise reconstruction objectives [27]. By decoupling perceptual compression from the generative process, our LDM framework effectively preserves these subtle anatomical textures while maintaining the diversity required for clinical plausibility.

This contribution is particularly timely given the current trajectory of the TMJ AI research community. To date, the majority of deep learning applications in this domain have predominantly concentrated on the automated segmentation of the mandibular condyle and glenoid fossa or the classification of OA stages [28,29,30]. However, a recurrent theme across these studies is the performance bottleneck attributed to small, single-center cohorts and the lack of external validation. Unlike other anatomical regions where massive public datasets accelerate innovation (e.g., chest X-rays or brain MRI), the development of robust, generalizable models for TMJ analysis has been stalled by the inability to share raw CBCT data containing sensitive facial biometrics.

By providing a mechanism to generate infinite, anonymous, and anatomically accurate synthetic samples, our work addresses this fundamental barrier. Unlike traditional de-identification methods that often require stripping the skull—and consequently removing the TMJ—our generative approach synthesizes “virtual patients” that retain the diagnostic region of interest while largely eliminating biometric information, as the model is trained exclusively on cropped 2D TMJ slices that exclude identifiable craniofacial features. This paradigm shift offers the community a potential alternative to the fragmented, private datasets that currently dominate the field, potentially standardizing benchmarks for future TMJ AI algorithms.

Data scarcity, particularly the lack of labeled pathological samples, is a primary bottleneck in developing robust diagnostic AI models for TMDs. The distribution of TMJ diseases is often long-tailed; severe osteoarthritis and specific rare deformities are under-represented in clinical datasets compared to healthy controls [31].

The diversity of CBCT images synthesized by *TMJCTGenerator* offers a promising avenue for data augmentation in downstream tasks [32]. By serving as an effective data sampler, the model can fill critical gaps within the training distribution. Specifically, by generating diverse variations of pathological features, such as varying severities of bone erosion that may be underrepresented in limited real-world datasets, *TMJCTGenerator* may be able to encourage downstream classifiers to learn more robust decision boundaries such as automatic OA classification or condylar segmentation. This potential is supported by recent findings in lung nodule detection [33] and brain MRI segmentation [34,35], which confirm that high-fidelity synthetic data can serve as a viable substitute or supplement to real data, particularly in privacy-sensitive medical applications [36]. However, we acknowledge that the downstream utility of our synthetic images has not been empirically validated in the current study; dedicated augmentation and transfer experiments are needed to confirm this potential. Moreover, synthetic data generated from a single-center cohort may amplify existing distributional biases, and careful validation against real-world populations would be necessary before using such data to train diagnostic models.

Our framework generates 2D sagittal and coronal slices rather than full 3D volumes. This design is justified by several factors: clinical TMJ OA diagnosis relies predominantly on these cross-sectional views [5]; current deep learning models in the TMJ field are likewise trained on 2D slices [28,29,37], making 2D synthetic images directly applicable to existing workflows; and the use of cropped 2D slices offers enhanced privacy protection by excluding identifiable craniofacial anatomy. However, generating 2D slices does not preserve volumetric consistency, which may limit applications requiring 3D reconstruction. Future research will focus on extending the architecture to 3D voxel grids or video-diffusion techniques to ensure inter-slice coherence. Furthermore, while the current study focuses on osseous changes via CBCT, the LDM framework is theoretically adaptable to soft-tissue assessment. Given the challenges of limited accessibility and resolution in TMJ MRI, our generative approach could be extended to synthesize high-resolution MRI sequences, thereby providing a more comprehensive synthetic dataset for TMJ diagnostics.

Several limitations of this study warrant discussion. First, our current framework synthesizes 2D sagittal or coronal slices. While diagnostic interpretation of TMJ OA heavily relies on these cross-sectional views, a complete CBCT examination is inherently 3D. Generating independent 2D slices ignores the volumetric consistency. Future research will focus on extending the LDM architecture to 3D voxel grids or employing video-diffusion techniques to ensure inter-slice coherence. Second, the current model generates images based on random noise or simple class labels. Integrating text-conditioning using large language models as encoders would allow for more controllable synthesis, enabling clinicians to generate specific pathological scenarios for training or research. Third, all data were collected from a single center, and no external validation was performed. The generalizability of the model to CBCT data acquired with different scanners, imaging protocols, or patient populations remains to be established in future multi-center studies. Fourth, only two dentists participated in the visual Turing test; future studies with a larger panel of evaluators are warranted.

## 5. Conclusions

In this study, we presented TMJCTGenerator, a novel framework based on LDMs for the high-fidelity synthesis of TMJ CBCT images. Our comprehensive evaluation demonstrates that LDMs effectively address the trade-off between image fidelity and diversity, significantly outperforming traditional VAE and GAN baselines. The generated images preserve critical osseous microstructures essential for the diagnosis of degenerative joint diseases and have potential for data augmentation in downstream tasks.

By providing a preliminary solution to the critical bottleneck of data scarcity and privacy concerns in oral radiology, this work may facilitate more robust and scalable TMJ and dental AI applications. To foster collaboration and reproducibility, we have open-sourced our code and pre-trained weights, offering the community a foundational tool for advanced TMJ image analysis and synthesis.

## Figures and Tables

**Table 1:** Demographic characteristics of the training and test datasets.

	Training Dataset	Test Dataset	*p*-Value
**Sex**			0.827 *
Male	64	6	
Female	284	33	
**Age**			0.732 ^#^
Mean (SD)	28.7 (13.1)	29.5 (13.4)	
Median (IQR)	25.4 (16.9)	27.3 (19.8)	

* Fisher exact test; ^#^ Wilcoxon rank sum test.

**Table 2:** Implementation details and hyperparameters for the proposed TMJCTGenerator.

Category	Parameter	Value/Description
Environment	GPU	NVIDIA RTX 5090 (24GB VRAM)
	Framework	PyTorch 2.8.0
	Precision	BFloat16
Data Processing	Input Resolution	256 ×256 pixels
	Normalization Range	$\left[-1,1\right]$
	Augmentation	Random Horizontal Flip (p=0.5), Affine (Rot $\pm 5^{\circ }$, Trans <5%), Color Jitter
Model Architecture	VAE Model	Pre-trained
	Latent Dimension	4×32×32
	U-Net Channels	{128, 256, 512, 512}
	Condition Embedding	Class Embedding
Training	Optimizer	AdamW
	Learning Rate	$1\times 10^{-4}$
	LR Scheduler	Cosine Annealing with Linear Warmup
	Batch Size	64
	Epochs	200
	Gradient Clipping	Norm = 1.0
Inference	Sampler	Denoising Diffusion Implicit Models
	Sampling Steps	50
	Guidance Scale	s=5.0

**Table 3:** Quantitative comparison of generative performance (FID scores) among different models.

	cDCGAN	cVAE	TMJCTGenerator
Coronal	798.147	3.669	2.918
Sagittal	901.265	5.425	3.125

**Table 4:** Mean differences in visual Turing scores between generated and real images in the non-inferiority test.

	Mean	95% CI Lower Bound	95% CI Upper Bound
**Coronal**			
Condyle	0.090	−0.070	0.251
TMJ	−0.060	−0.305	0.193
**Sagittal**			
Condyle	−0.120	−0.274	0.035
TMJ	−0.350	−0.659	−0.033

## Data Availability

The data presented in this study are available on request from the corresponding author due to ethical reasons. The source code and pre-trained models of TMJCTGenerator have been made open-source at https://github.com/Zheng-Yunhao/TMJCTGenerator (accessed on 24 February 2026).
